# ﻿Review of the genus *Hauptenia* Szwedo (Hemiptera, Fulgoromorpha, Derbidae), with descriptions of two new species from China

**DOI:** 10.3897/zookeys.1157.97646

**Published:** 2023-04-03

**Authors:** Yong-Jin Sui, Lin Yang, Jian-Kun Long, Zhi-Min Chang, Xiang-Sheng Chen

**Affiliations:** 1 Institute of Entomology, Guizhou University, Guiyang, Guizhou, 550025, China; 2 The Provincial Special Key Laboratory for Development and Utilization of Insect Resources of Guizhou, Guizhou University, Guiyang, Guizhou, 550025, China; 3 Guizhou Key Laboratory for Plant Pest Management of Mountainous Region, Guizhou University, Guiyang, Guizhou, 550025, China

**Keywords:** Cedusini, distribution, new record, planthoppers, taxonomy

## Abstract

The derbid planthopper genus *Hauptenia* Szwedo, 2006 is reviewed. Two new species from China, *H.beibengensis* Sui & Chen, **sp. nov.** and *H.daliensis* Sui & Chen, **sp. nov.**, are described and illustrated. A third species, *H.tripartita*Rahman et al., 2012, is recorded from China for the first time. An updated checklist and identification key to all ten known species of the genus *Hauptenia* are provided.

## ﻿Introduction

The planthopper family Derbidae (Hemiptera, Fulgoromorpha) was established by Spinola in 1839, containing 22 tribes in three subfamilies (Bourgoin 2023), eight tribes, 38 genera, and 156 species of which are known in China (Yang and Wu 1994; Sui and Chen 2019; Bourgoin 2023). Most of these taxa are distributed in the Oriental bioregion, especially in southern China.

In the Breddiniolinae Fennah, 1950, Cedusini Emeljanov, 1992 and subtribe Cedusina Emeljanov, 2008, the genus *Hauptenia* was established by Szwedo (2006) for five Chinese species (previously in *Malenia* Haupt, 1924) and with *Maleniamagnifica* Yang & Wu, 1994 as its type species. Rahman et al. (2012) described two Korean species and Jhan et al. (2016) described one Bangladeshi species, bringing the total of known species to eight: *H.bandarbanensis* Jhan & Rahman, 2016, *H.fellea* (Yang & Wu, 1994), *H.glutinosa* (Yang & Wu, 1994), *H.idonea* (Yang & Wu, 1994), *H.jacula* (Yang & Wu, 1994), *H.magnifica* (Yang & Wu, 1994), *H.palgongsanensis* Rahman, Kwon & Suh, 2012, and *H.tripartita* Rahman, Kwon & Suh, 2012.

Herein, two new species, *Haupteniabeibengensis* Sui & Chen, sp. nov. and *H.daliensis* Sui & Chen, sp. nov., are described and illustrated from China, bringing the total number of this genus to ten. One species, *H.tripartita*Rahman et al., 2012, is recorded from China for the first time, and a key is provided to all ten species of *Hauptenia*.

## ﻿Materials and methods

The morphological terminology follows Bourgoin (1987), Bourgoin and Huang (1990) and Yang and Wu (1994). Body length was measured from apex of vertex to tip of fore wing by KEYENCE VHX-1000E system. The standard terminology of venation follows Bourgoin et al. (2015). Dried specimens were used for the descriptions and illustrations. External morphology was observed under a stereoscopic microscope and all measurements were done with an ocular micrometer. Color pictures for adult habitus were obtained by the KEYENCE VHX-6000 system. Genital segments were macerated in 10% NaOH and drawn from preparations in glycerin jelly using a Leica MZ 12.5 stereomicroscope. Illustrations were scanned with a Canon CanoScan LiDE 220 and imported into Adobe Photoshop CS5 for labeling and plate composition. The dissected genitalia were preserved in glycerin in small plastic tubes pinned together with the specimens.

The type specimens and examined specimens are deposited in the Institute of Entomology, Guizhou University, Guiyang, Guizhou Province, China (**GUGC**).

## ﻿Taxonomy

### ﻿Taxonomy of the genus Hauptenia

Taxon classificationAnimaliaHemipteraDerbidae

Szwedo, 2006

D8903B9C-FEA5-5E40-8B20-433933A6FFD4

Figs 1–4 , 5–13 , 14–22

Hauptenia Szwedo, 2006: 331–332; Rahman et al. 2012: 63; Jhan et al. 2016: 2.

#### Type species.

*Maleniamagnifica* Yang & Wu, 1994: by original designation.

#### Diagnostic characters.

**Figures 1–4.**
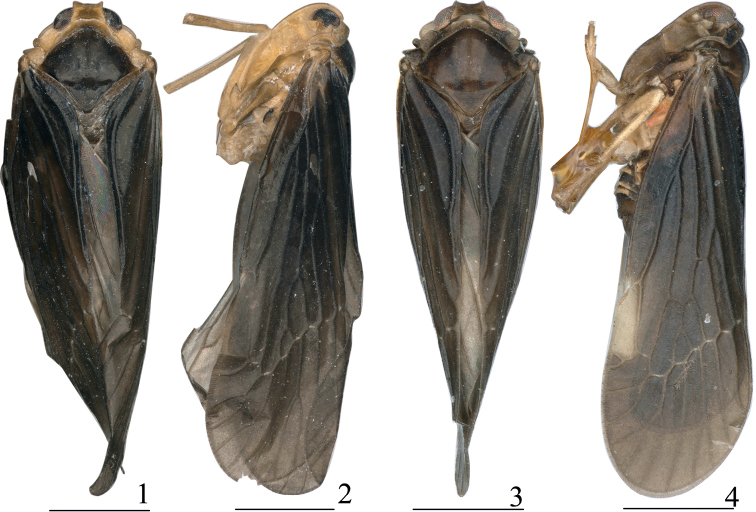
Male habitus (dorsal and lateral views) **1, 2***Haupteniabeibengensis* Sui & Chen, sp. nov. **3, 4***Haupteniadaliensis* Sui & Chen, sp. nov. Scale bars: 1 mm.

**Figures 5–13.**
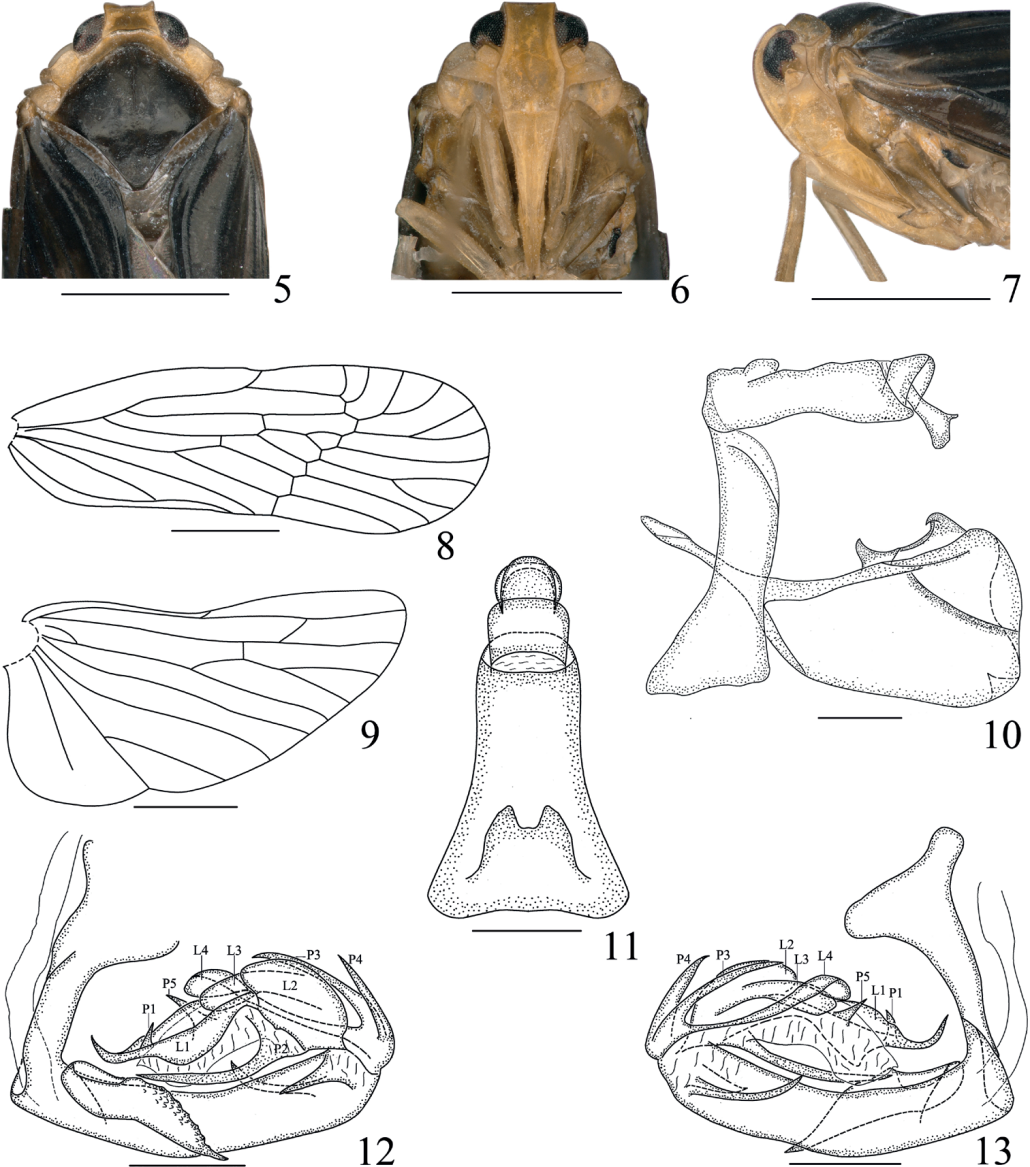
*Haupteniabeibengensis* Sui & Chen, sp. nov., male **5** head and thorax, dorsal view **6** face **7** head and thorax, left lateral view **8** fore wing **9** hind wing **10** terminalia, left lateral view **11** anal tube, dorsal view **12** phallic complex, left lateral view **13** phallic complex, right lateral view. Scale bars: 1 mm (**5–9**); 0.2 mm (**10–13**).

**Figures 14–22.**
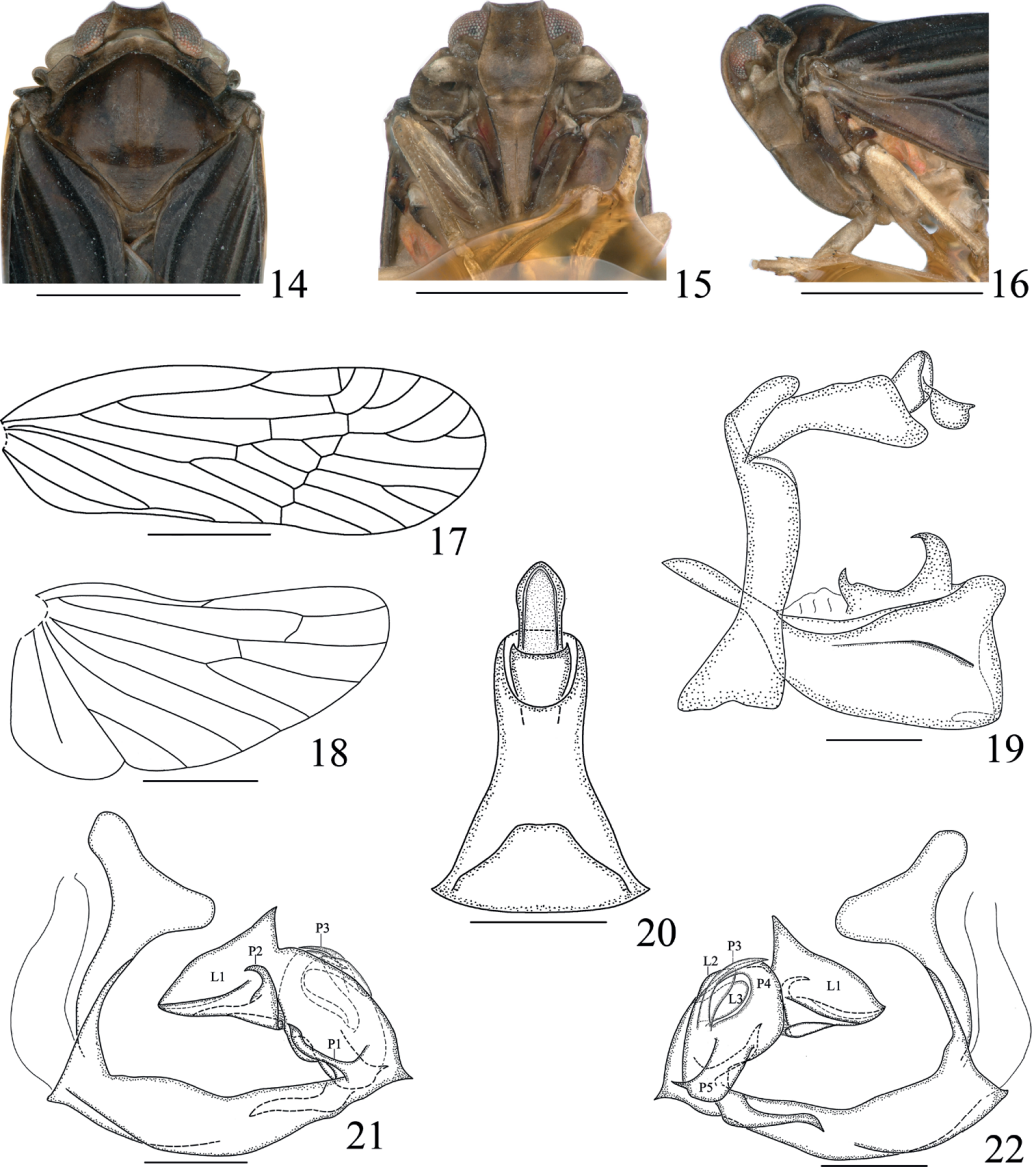
*Haupteniadaliensis* Sui & Chen, sp. nov., male **14** head and thorax, dorsal view **15** face **16** head and thorax, left lateral view **17** fore wing **18** hind wing **19** terminalia, left lateral view **20** anal tube, dorsal view **21** phallic complex, left lateral view **22** phallic complex, right lateral view. Scale bars: 1 mm (**14–18**); 0.2 mm (**19–22**).

Combination of the following characters: head (Figs 1, 3, 5, 14) with eyes distinctly narrower than pronotum. Vertex (Figs 1, 3, 5, 14) trapezoidal, at base slightly wider than at apex, slightly projecting in front of eyes. Frons (Figs 6, 15) longer in middle line than widest part ~ 1.48–1.84: 1 and shorter than clypeus ~ 1: 1.1–1.53, without median carina. Antennae (Figs 6, 7, 15, 16) short, subantennal process well developed. Fore wing (Figs 8, 17) longer than widest part ~ 2.6–3.1: 1, RA with one or two terminal(s). Hind wing (Figs 9, 18) with vein ScP+RA very short, CuA with two or three terminals; spinal formula of hind leg 7–6–5. Male terminalia (Figs 10, 19) with gonostyli symmetrical, short and stout, dorsobasal projection distad; pygofer with dorsocaudal angle not produced into finger-shaped process; anal tube not distinctly elongated, dorsal margin shorter than ventral margin in lateral view, usually with apex not reaching level of apex of gonostyli, epiproct turned ventrad or nearly so, slightly notched at apex in lateral view.

##### ﻿Checklist and distributions of species of *Hauptenia* Szwedo, 2006

*H.bandarbanensis* Jhan & Rahman, 2016; Bangladesh.

*H.beibengensis* Sui & Chen, sp. nov.; China (Xizang).

*H.daliensis* Sui & Chen, sp. nov.; China (Yunnan).

*H.fellea* (Yang & Wu, 1994); China (Guizhou, Sichuan, Taiwan, Yunnan).

*H.glutinosa* (Yang & Wu, 1994); China (Chongqing, Fujian, Guizhou, Hainan, Hunan, Taiwan, Zhejiang).

*H.idonea* (Yang & Wu, 1994); China (Guizhou, Taiwan).

*H.jacula* (Yang & Wu, 1994); China (Guangxi, Guizhou, Hainan, Taiwan).

*H.magnifica* (Yang & Wu, 1994); China (Guangxi, Guizhou, Hainan, Taiwan, Yunnan).

*H.palgongsanensis* Rahman, Kwon & Suh, 2012; Korea.

*H.tripartita* Rahman, Kwon & Suh, 2012; Korea; China (Anhui, Guangxi, Guizhou, Hunan, Liaoning, Shaanxi, Sichuan, Zhejiang); new record for China.

### ﻿Key to the species of genus *Hauptenia* Szwedo, 2006 (based on Jhan et al. 2016)

**Table:** 

1	Gonostyli with dorsocaudal angle produced into finger-shaped process (Fig. 19; Rahman et al. 2012: fig. 20)	**2**
–	Gonostyli with dorsocaudal angle not produced into finger-shaped process (Fig. 10; Rahman et al. 2012: fig. 31)	**6**
2	Gonostyli with apical hook of dorsobasal projection quadrate, apical margin obliquely truncate (Rahman et al. 2012: fig. 20)	**3**
–	Gonostyli with apical hook of dorsobasal projection triangular, apical margin truncate (Fig. 19; Yang and Wu 1994: fig. 42E)	**4**
3	Endosoma of aedeagus (Rahman et al. 2012: figs 17, 18) with four lobes and four processes, length of middle processes ca. half of left and right processes; mesonotum dark brown to black	***H.palgongsanensis* Rahman et al., 2012**
–	Endosoma of aedeagus (Yang and Wu 1994: fig. 41H, I) with two lobes and four processes, middle processes as long as left and right processes; mesonotum yellow	***H.fellea* (Yang & Wu, 1994)**
4	General color dark brown; hind wing (Fig. 18) with vein CuA with two terminals	***H.daliensis* Sui & Chen, sp. nov.**
–	General color yellowish brown; hind wing (Yang and Wu 1994: fig. 42D) with vein CuA with three terminals	**5**
5	Endosoma of aedeagus (Yang and Wu 1994: figs 42H, I) with one large lobe and five processes; body relatively small, body length including fore wing male 3.9–4.3 mm, female 4.9 mm	***H.glutinosa* (Yang & Wu, 1994)**
–	Endosoma of aedeagus (Yang and Wu 1994: figs 43G, H) with one large lobe and four processes; body relatively large, body length including fore wing male 5.4–5.7 mm, female 5.9 mm	***H.idonea* (Yang & Wu, 1994)**
6	Hind wing with CuA with three terminals (Fig. 9); gonostyli (Fig. 10) with each inner lower surface with a small hook apically	**7**
–	Hind wing with CuA with two terminals (Yang and Wu 1994: fig. 40D); gonostyli with each inner lower surface without hook apically	***H.jacula* (Yang & Wu, 1994)**
7	General color dark brown; gonostyli with apical margin near truncate (Fig. 10)	***H.beibengensis* Sui & Chen, sp. nov.**
–	General color yellow to yellowish brown; gonostyli with apical margin obliquely truncate (Rahman et al. 2012: fig. 31)	**8**
8	Endosoma of aedeagus (Rahman et al. 2012: figs 28, 29) with four lobes, longest one wide and tripartite	***H.tripartita* Rahman et al., 2012**
–	Endosoma of aedeagus with four lobes, longest one slender and monopartite	**9**
9	Endosoma of aedeagus (Yang and Wu 1994: figs 39H, I) with five processes, and two lobes out of four produced into processes	***H.magnifica* (Yang & Wu, 1994)**
–	Endosoma of aedeagus (Jhan et al. 2016: figs 1H, I) with six processes, and three lobes out of four produced into processes	***H.bandarbanensis* Jhan & Rahman, 2016**

### ﻿Descriptions

### ﻿ Hauptenia bandarbanensis

Taxon classificationAnimaliaHemipteraDerbidae

Jhan & Rahman, 2016

550AABE2-5EB1-59C5-B346-0DD8AFD55425

Hauptenia bandarbanensis Jhan & Rahman, in Jhan et al. 2016: 2–3, figs 1, 2.

#### Material examined.

No specimen examined.

#### Diagnostic characters.

(Based on Jhan et al. 2016). General color yellowish brown. Fore wing light brown, hind wing pale brown. Fore wing longer than widest part ~ 3: 1, RA with two terminals, MP with four sectors. Hind wing with CuA with three terminals. Gonostyli with apical margin obliquely truncate, dorsocaudal angle not produced; each inner lower surface with a hook subapically. Endosoma of aedeagus with six processes and three lobes out of four produced into processes.

#### Distribution.

Bangladesh.

### ﻿ Hauptenia beibengensis

Taxon classificationAnimaliaHemipteraDerbidae

Sui & Chen sp. nov.

900DE357-5386-5998-A138-0C13CDC6380F

https://zoobank.org/43354BDE-7248-4623-9A54-0D671318F12D

Figs 1 , 2 , 5–13

#### Type material.

***Holotype***, ♂, **China**: Xizang, Motuo, Beibeng (29.25°N, 95.18°E), 15 August 2020, Y-J Sui. Paratypes, 3♂, same data as holotype.

#### Measurements.

Body length (including fore wing): male 4.86–5.01 mm (*n* = 4); fore wing length: male 4.45–4.57 mm (*n* = 4).

#### Description.

***Coloration*.** General color dark brown. Vertex (Figs 1, 5), frons (Fig. 6), gena (Figs 2, 7), antennae, subantennal process, ocelli, pronotum and tegula (Figs 1, 5, 7) yellowish brown. Rostrum brown, with apex fuscous. Eyes (Figs 1, 2, 5–7) slightly dark brown. Ocelli (Figs 2, 7) slightly yellowish white. Mesonotum (Figs 1, 5) dark brown, with median carina slightly lighter. Fore wing (Figs 1, 2) dark brown, veins same color. Hind wing subhyaline, brownish, veins lightly darker. Thorax with ventral areas yellowish brown. Legs brownish yellow. Genital segment dark brown.

***Head and thorax*.** Head (Figs 1, 5) including eyes distinctly narrower than pronotum (1: 1.49), short. Vertex (Figs 1, 5) trapezoidal, length between basal angles wider than length in middle line (2.42: 1), slightly projecting in front of eyes, posterior margin slightly concave, lateral carinae slightly elevated, median carina absent, disk slightly depressed. Frons (Fig. 6) longer in middle line than at the widest parts (1.54: 1), shorter than clypeus (1: 1.51), near apical 2/5 widest, disk depressed in entire length, lateral carinae keeled. Clypeus (Fig. 6) distinctly carinate medially from near basal 1/3. Apical segment of rostrum longer than wide. Antennae (Figs 2, 6, 7) short, second antennomere oval, flagellum originated from apical point. Subantennal processes (Figs 2, 7) distinct, ear-shaped. Transversely oblique carina across the gena between subantennal process and lateral carina of frons distinct. Eyes (Figs 5–7) semicircular. Lateral ocelli distinct, adjacent to eyes and antennae. Median length of pronotum distinctly less than that of vertex, anterior margin between eyes broadly convex, length behind eyes as long as median length. Mesonotum dorsally elevated, in lateral view raised above vertex, with median carinae reaching to the middle, posterior end triangularly depressed. Fore wing (Fig. 8) narrow, ~ 3.1× as long as the widest point, clavus closed, RA with two terminals, MP with four sectors. Hind wing (Fig. 9) shorter than fore wing, with RP reaching to apical margin, CuA with three terminals. Hind tibia without lateral spine. Spinal formula of hind leg 7–6–5.

***Male terminalia*.** Anal tube (Fig. 10) moderately long, in dorsal view, lateral margin narrowed gradually toward the near middle and then parallel toward apex, width at base larger than the narrowest part ~ 1.9: 1, length in middle line (including epiproct) than widest part at base ~ 1.75: 1, dorsolateral margin convex medially near base; epiproct turned ventrad. Pygofer (Fig. 10) in lateral view distinctly shorter dorsally than ventrally, dorsocaudal angle not produced. Gonostyli (Fig. 10) symmetrical, short and stout, apical margin truncate, dorsocaudal angle not produced; each inner lower surface with small triangular process apically; inner side of laterodorsal margin with broad projection distad, in left lateral view, basal hook shorter and stout, apical hook slightly turned outward at end. Phallic complex (Figs 12, 13) asymmetrical. Periandrium slightly curved, in left view, a big process arising from dorsal margin at base, and a short process arising from end with apex acute; in right view, a long and slender process arising from end near ventral margin. Endosoma more complex, with four lobes, one membrane and five processes of different sizes. Among four lobes, the longest lobe (L1) produced reaching to near base of periandrium, acute at apex; another other three lobes (L2–L4) round at apex, close together. In left lateral view, a small process (P1) arising from the longest lobe near apex, acute at apex; a long process (P2) arising from ventral margin of endosoma near at base, reaching to middle of periandrium; two long and sharped processes (P3, P4) arising from dorsal margin at base, pointed cephalad. In right lateral view, a small triangular process (P5) arising from the membrane one at base near dorsal margin.

#### Remarks.

This species is similar to *H.fellea* (Yang & Wu, 1994), but differs from the latter in the mesonotum (Figs 1, 5) dark brown with median carinae reaching to the middle (mesonotum yellowish brown with median carinae reaching to near end in *H.fellea*); gonostyli (Fig. 10) with dorsocaudal angle not produced (gonostyli with dorsocaudal angle produced into finger-shaped process in *H.fellea*); endosoma (Figs 12, 13) with four lobes, one membrane and five processes of different sizes (endosoma with two lobes and four processes in *H.fellea*).

#### Etymology.

This species is named after the collection site of the holotype, Beibeng Township in Xizang.

#### Host plants.

Unknown.

#### Distribution.

China (Xizang).

### ﻿ Hauptenia daliensis

Taxon classificationAnimaliaHemipteraDerbidae

Sui & Chen sp. nov.

73BEEC5A-B8CD-52BE-ABEE-463AC3ED8709

https://zoobank.org/F6ECE0A6-154E-4F8A-BBF6-A49655AF95CA

Figs 3 , 4 , 14–22

#### Type material.

***Holotype***, ♂, **China**: Yunnan, Dali, Mt. Cangshan (25.67°N, 100.13°E), 18 June 2009, B. Li & Z-H. Yang. Paratypes, 3♂, same data as holotype.

#### Measurements.

Body length (including fore wing): male 4.56–4.71 mm (*n* = 4); fore wing length: male 4.02–4.17 mm (*n* = 4).

#### Description.

***Coloration*.** General color dark brown. Vertex (Figs 3, 14), gena (Figs 4, 16), antennae, subantennal process, pronotum and tegula slightly lighter. Frons (Fig. 15) and clypeus with lateral margin dark. Rostrum brown, with apex fuscous. Eyes (Figs 3, 4, 14–16) slightly dark red. Ocelli (Figs 4, 16) yellowish white. Mesonotum (Figs 3, 14) brown, with median carina slightly lighter. Fore wing (Figs 3, 4) dark brown except cell of ScP lighter, veins concolor. Hind wing subhyaline, brownish, veins lightly darker. Thorax with ventral areas yellow to orange red. Legs brownish yellow. Genital segment dark brown.

***Head and thorax*.** Head (Figs 3, 14) including eyes distinctly narrower than pronotum (1: 1.44), short. Vertex (Figs 3, 14) trapezoidal, length between basal angles wider than length in middle line (3.6: 1), slightly projecting in front of eyes, posterior margin concave, lateral carinae slightly elevated, median carina absent, disk slightly depressed. Frons (Fig. 15) longer in middle line than at the widest parts (1.45: 1), shorter than clypeus (1: 1.48), near apical 2/5 widest, disk depressed in entire length, lateral carinae keeled. Clypeus distinctly carinate medially from near base 1/3. Apical segment of rostrum longer than wide. Antennae (Figs 4, 15, 16) short, second antennomere subglobose, flagellum originated from apical point. Subantennal processes distinct, ear-shaped. Transversely oblique carina across the gena between subantennal process and lateral carina of frons distinct. Eyes (Figs 3, 4, 14–17) semicircular. Lateral ocelli (Figs 4, 16) distinct, adjacent to eyes and antennae. Median length of pronotum distinctly less than that of vertex, anterior margin between eyes broadly convex, length behind eyes slightly greater than median length. Mesonotum (Figs 3, 14) dorsally elevated, in lateral view raised above vertex, with median carinae reaching to the apical 1/3, posterior end triangularly depressed. Fore wing (Fig. 17) narrow, ~ 3× as long as at the widest point, clavus closed, RA with one or two terminal(s), MP with four sectors. Hind wing (Figs 18) shorter than fore wing, with RP reaching to apical margin, CuA with two terminals. Hind tibia without lateral spine. Spinal formula of hind leg 7–6–5.

***Male terminalia*.** Anal tube (Fig. 19) moderately long, in dorsal view, lateral margin narrowed gradually toward the near middle and then wider slightly toward apex, width at base larger than the narrowest part ~ 2.5: 1, length in middle line (including epiproct) than widest part at base ~ 1.62: 1, dorsolateral margin convex medially near base; epiproct turned ventrad. Pygofer (Fig. 19) in lateral view distinctly shorter dorsally than ventrally, dorsocaudal angle not produced. Gonostyli (Fig. 19) symmetrical, short and stout, apical margin truncate, dorsocaudal angle produced into finger-shaped process; each inner lower surface with small hook apically; inner side of laterodorsal margin with broad projection distad, in left lateral view, left hook shorter and smaller than apical hook, apical hook slightly turned outward at end. Phallic complex (Figs 21, 22) asymmetrical. Periandrium curved, with a small acute process at end, visible in both left and right lateral views; in right view, a strong process arising from near end, wavy and parallel with periandrium approximately. Endosoma complex, with three lobes and five processes of different sizes. Among three lobes, in left lateral view, the largest lobe (L1) with dorsal margin produced into triangular process near apical 1/3, and with keel from apex to near apical 1/3 near ventral margin; in right lateral view, the largest lobe ventral margin rolling up at third of apex, another two small lobes (L2, L3) arising from ca. middle of endosoma dorsally. In left lateral view, a long and slender process (P1) arising from basal of endosoma, a process (P2) arising from the ventral margin of the largest lobe near apical 2/5, curved and pointed cephalad; and in right lateral view, a slender process (P3) and a process (P4) broad at base, all curved at dorsal margin, pointed oppositely; another process (P5) arising from ca. middle of endosoma ventrally, abruptly narrowed subapically, acute at apex, pointed caudally.

#### Remarks.

This species is similar to *H.fellea* (Yang & Wu) but differs from the latter in the hind wing (Fig. 18) with CuA with two terminals (CuA with three terminals in *H.fellea*); gonostyli (Fig. 19) with apical margin truncate (gonostyli with apical margin obliquely truncate in *H.fellea*); endosoma (Figs 21, 22) with three lobes and five processes (endosoma with two lobes and four processes in *H.fellea*).

#### Etymology.

This species is named after the collection site of the holotype, Dali City in Yunnan.

#### Host plants.

Unknown.

#### Distribution.

China (Yunnan).

### ﻿ Hauptenia fellea

Taxon classificationAnimaliaHemipteraDerbidae

(Yang & Wu, 1994)

9604A9FF-CA6A-5A0B-90F6-1B6FE51829E8

Malenia fellea Yang & Wu, 1994: 89, fig. 41.
Hauptenia fellea (Yang & Wu, 1994): Szwdeo 2006: 331.

#### Material examined.

**China**: 5♂♂, Guizhou, Zhijin, 21 June 2019, Z-C Zhou; 4♂♂, Sichuan, Dayi, 20 July 2022, Y-J Sui; 1♂, Yunnan, Mengla, 13 November 2018, L-K Zhong.

#### Diagnostic characters.

General color brown. Fore wing black, hind wing grayish. Fore wing longer than widest part ~ 2.7: 1, RA with two terminals, MP with four sectors. Hind wing with CuA with three terminals. Gonostyli with apical margin obliquely truncate, dorsocaudal angle produced into finger-shaped process; each inner lower surface without hook subapically. Endosoma of aedeagus with four processes and two elongated lobes.

#### Distributions.

China (Guizhou, Sichuan, Taiwan, Yunnan).

### ﻿ Hauptenia glutinosa

Taxon classificationAnimaliaHemipteraDerbidae

(Yang & Wu, 1994)

AA4C3664-A006-5492-B000-F1A0D3BA3316

Malenia glutinosa Yang & Wu, 1994: 91, fig. 42.
Hauptenia glutinosa (Yang & Wu, 1994): Szwedo 2006: 331.

#### Material examined.

**China**: 7♂♂, Chongqing, Beibei, Mt. Jinyun, 12 July 2021, Y-J Sui; 1♂, Fujian, Jian’ou, 25 August 2019, Z-C Zhou; 2♂♂, Guizhou, Suiyang, 26 June 2019, Y-J Sui; 1♂, Hainan, Lingshui, 17 July 2007, Z-G Zhang; 2♂♂, Hunan, Yongshun, 21 August 2016, L-J Yang and Y-S Ding; 2♂♂, Zhejiang, Lin’an, Mt. Tianmu, 20 July 2009, Y Chen and Z-H Meng.

#### Diagnostic characters.

General color yellow. Fore wing pale brown, hind wing grayish. Fore wing longer than widest part ~ 2.7: 1, RA with two terminals, MP with four sectors. Hind wing with CuA with three terminals. Gonostyli with apical margin truncate, dorsocaudal angle produced into finger-shaped process; each inner lower surface without hook subapically. Endosoma of aedeagus with one large lobe and five processes.

#### Distributions.

China (Chongqing, Fujian, Guizhou, Hainan, Hunan, Taiwan, Zhejiang).

### ﻿ Hauptenia idonea

Taxon classificationAnimaliaHemipteraDerbidae

(Yang & Wu, 1994)

4EA4E304-304B-5A54-877B-3D87F51FF73F

Malenia idonea Yang & Wu, 1994: 94, fig. 43.
Hauptenia idonea (Yang & Wu, 1994): Szwedo 2006: 332.

#### Material examined.

**China**: 1♂, Guizhou, Leishan, Mt. Leigong, 10 July 2011, W-B Zheng; 1♂, Taiwan, Gaoxiong, 21 November 2002, X-S Chen.

#### Diagnostic characters.

General color deep yellow. Fore wing light black, hind wing grayish. Fore wing longer than widest part ~ 2.7: 1, RA with two terminals, MP with four sectors. Hind wing with CuA with three terminals. Gonostyli with apical margin truncate, dorsocaudal angle produced into finger-shaped process; each inner lower surface without hook subapically. Endosoma of aedeagus with one large lobe and four processes.

#### Distributions.

China (Guizhou, Taiwan).

### ﻿ Hauptenia jacula

Taxon classificationAnimaliaHemipteraDerbidae

(Yang & Wu, 1994)

A4EF4E11-F59C-583D-84A6-766D7715D854

Malenia jacula Yang & Wu, 1994: 89, fig. 40.
Hauptenia jacula (Yang & Wu, 1994): Szwedo 2006: 331.

#### Material examined

**. China**: 1♂, Guangxi, Longsheng, 14 May 2021, M Deng; 1♂, Guizhou, Jiangkou, 25 May 2021, Y-J Sui; 1♂, Guizhou, Duyun, 9 June 2017, L-J Yang; 1♂, Hainan, Changjiang, 26 April 2021, Y-J Sui.

#### Diagnostic characters.

General color yellow. Fore wing pale brown, hind wing grayish. Fore wing longer than widest part ~ 2.6: 1, RA with one terminal, MP with four sectors. Hind wing with CuA with two terminals. Gonostyli with apical margin truncate, dorsocaudal angle not produced; each inner lower surface without hook subapically. Endosoma of aedeagus with three processes and two lobes.

#### Distributions.

China (Guangxi, Guizhou, Hainan, Taiwan).

### ﻿ Hauptenia magnifica

Taxon classificationAnimaliaHemipteraDerbidae

(Yang & Wu, 1994)

A6EAEA09-14CF-5BE7-9597-7F7B19CB9EF6

Malenia magnifica Yang & Wu, 1994: 86, fig. 39.
Hauptenia magnifica (Yang & Wu, 1994): Szwedo 2006: 331.

#### Material examined.

**China**: 2♂♂, Guangxi, Huanjiang, 27 July 2019, Y-J Sui; 5♂♂, Guizhou, Wangmo, 29 June 2013, J-C Xing; 1♂, Hainan, Ledong, 12 July 2007, Q-Z Song; 1♂, Yunnan, Mengla, 29 August 2017, Y Zhi.

#### Diagnostic characters.

General color yellow. Fore wing pale brown, hind wing dirty white. Fore wing longer than widest part ~ 2.7: 1, RA with two terminals, MP with four sectors. Hind wing with CuA with three terminals. Gonostyli with apical margin obliquely truncate, dorsocaudal angle not produced; each inner lower surface with a hook subapically, directed basad. Endosoma of aedeagus with five processes, and two lobes out of four produced into processes.

#### Distributions.

China (Guangxi, Guizhou, Hainan, Taiwan, Yunnan).

### ﻿ Hauptenia palgongsanensis

Taxon classificationAnimaliaHemipteraDerbidae

Rahman, Kwon & Suh, 2012

C00FE443-0C8E-574B-B349-1072D47DCE4F

Hauptenia palgongsanensis Rahman, Kwon & Suh, 2012: 65, figs 12–22.

#### Material examined.

No specimen examined.

#### Diagnostic characters.

(Based on Rahman et al. 2012). General color dark brown. Fore wing dark brown to black, hind wing grayish white. Fore wing longer than widest part ~ 2.8: 1, RA with two terminals, MP with four sectors. Hind wing with CuA with three terminals. Gonostyli with apical margin obliquely truncate, dorsocaudal angle produced into finger-shaped process; each inner lower surface without hook subapically. Endosoma of aedeagus with four processes and four lobes.

#### Distribution.

Korea.

### ﻿ Hauptenia tripartita

Taxon classificationAnimaliaHemipteraDerbidae

Rahman, Kwon & Suh, 2012

44D7AD62-C0CA-5F49-B5D9-048FEE2C5375

Hauptenia tripartita Rahman, Kwon & Suh, 2012: 66, figs 23–33.

#### Material examined.

**China**: 1♂, Anhui, Jinzhai, Tianma National Nature Reserve, 27 June 2013, B Li and B Yan; 2♂♂, Guangxi, Xing’an, 23 July 2015, Q Luo; 3♂♂, Guizhou, Liping, 14 July 2016, Y-J Wang; 1♂, Hunan, Wugang, 11 August 2007, X-S Chen; 2♂♂, Liaoning, Kuandian, 31 August 2010, B Li; 2♂♂, Shaanxi, Foping, 4–9 August 2010, P Zhang; 3♂♂, Sichuan, Yingjing, 28 July 2022, F-E Li; 1♂, Zhejiang, Pan’an, 2 July 2013, B Li.

#### Diagnostic characters.

General color yellowish brown. Fore wing yellowish brown, hind wing grayish white. Fore wing longer than widest part ~ 2.7–2.9: 1, RA with two terminals, MP with four sectors. Hind wing with RP CuA with three terminals. Gonostyli with apical margin obliquely truncate, dorsocaudal angle not produced; each inner lower surface with a hook subapically, directed basad. Endosoma of aedeagus with six processes and four lobes, the largest lobe wide and tripartite.

#### Distributions.

China (Anhui, Guangxi, Guizhou, Hunan, Liaoning, Shaanxi, Sichuan, Zhejiang), Korea.

#### Note.

This species is recorded from China for the first time.

## ﻿Discussion

The genus *Hauptenia* Szwedo, 2006, belongs to the tribe Cedusini (Hemiptera: Derbidae: Breddiniolinae), which is characterized by sensory pits on head and on wings absent, subantennal process well developed, jugal margin of hind wings without stridulatory plate, and tibia of hind leg without lateral spine (Emeljanov 1996). The tribe Cedusini comprises the subtribes Cedusina Emeljanov, 1992 and Eocenchreina Emeljanov, 2008. The obvious difference between them is that species of Cedusina have cixiid venation of the clavus (fore wing with joined claval veins Pcu + A1 reaching commissural margin of fore wing, reaching vein A2), and species of Eocenchreina have achilid venation of the clavus (fore wing with joined claval veins Pcu +A1 reaching claval suture, reaching CuP, near apex of clavus). As the subtribe Eocenchreina was erected for Cedusini with the achilid venation of the clavus, the genus *Hauptenia* was indirectly placed in the subtribe Cedusina by Emeljanov (2008). Morphologically, the whole subtribe Cedusina are very similar externally, but *Hauptenia* may be easily distinguished from other genera of Cedusina by the short and stout gonostyli, the pygofer with its dorsocaudal angle not produced, and the spinal formula of the hind leg 7–6–5 (Szwedo 2006; Rahman et al. 2012). In terms of geographical distribution, *Hauptenia* may be closely related to *Produsa* and *Muiredusa* in the same subtribe Cedusina. However, for the exact relationships within the subtribe Cedusina, more specimens need to be examined and molecular biology techniques to be used in the future studies.

Due to the original literature not recording host plants of these planthoppers, they are not known. In our study, we found that a few specimens of *Hauptenia* (*H.fellea*, *H.magnifica*, and *H.tripartita*) were collected on bamboo. In addition, some specimens of *H.glutinosa* and *H.jacula* were collected by light traps, and we speculate that some species of the genus *Hauptenia* have positive phototropism.

Based on the diverse natural environment in China, we expect that further collecting will increase the number of new records or species, and suggest that specimens already collected and stored in collections should be reanalyzed.

## Supplementary Material

XML Treatment for Hauptenia

XML Treatment for Hauptenia bandarbanensis

XML Treatment for Hauptenia beibengensis

XML Treatment for Hauptenia daliensis

XML Treatment for Hauptenia fellea

XML Treatment for Hauptenia glutinosa

XML Treatment for Hauptenia idonea

XML Treatment for Hauptenia jacula

XML Treatment for Hauptenia magnifica

XML Treatment for Hauptenia palgongsanensis

XML Treatment for Hauptenia tripartita

## References

[B1] Bourgoin T (1987) A new interpretation of the homologies of the Hemiptera male genitalia, illustrated by the Tettigometridae (Hemiptera, Fulgoromorpha). Proceedings 6^th^Auchenorrhyncha Meeting, Turin, Italy, 7–11 September 1987, 113–120.

[B2] Bourgoin T (2023) FLOW (Fulgoromorpha Lists on The Web): a world knowledge base dedicated to Fulgoromorpha. Version 8. http://hemiptera-databases.org/flow/ [Updated 21 March 2023]

[B3] Bourgoin T Huang J (1990) Morphologie comparée des genitalia mȃles des Trypetimorphini et remarques phylogénétiques (Hemiptera: Fulgoromorpha: Tropiduchidae). Annales de la Société Entomologique de France. Nouvelle Série 26(4): 555–564.

[B4] Bourgoin T Wang RR Asche M Hoch H Soulier-Perkins A Stroiński A Yap S Szwedo J (2015) From micropterism to hyperpterism: recognition strategy and standardized homology-driven terminology of the fore wing venation patterns in planthoppers (Hemiptera: Fulgoromorpha). Zoomorphology 134: 63–77. 10.1007/s00435-014-0243-6 25705075 PMC4326643

[B5] Emeljanov AF (1996) On the system and phylogeny of the family Derbidae (Homoptera, Cicadina). Entomological Review 75(2): 70–100.

[B6] Emeljanov AF (2008) Two new genera of the family Derbidae from the New World, with description of recent and extinct Miocene new species (Homoptera, Fulgoroidea). Entomological Review 88(8): 910–915. 10.1134/S0013873808080046

[B7] Jhan PK Rahman MA Khan MH Jahan H (2016) A newly recorded genus *Hauptenia* Szwedo (Hemiptera: Fulgoromorph: Derbidae) in Bangladesh, with description of a new species. International Journal of Innovative Research 1(1): 1–4.

[B8] Rahman MA Kwon YJ Suh SJ (2012) Two newly recorded genera and three new species of the tribe Cedusini (Hemiptera: Fulgoromorpha: Derbidae) from Korea. Zootaxa 3261: 59–68. 10.11646/zootaxa.3261.1.3

[B9] Sui YJ Chen XS (2019) Review of the genus *Vekunta* Distant from China, with descriptions of two new species (Hemiptera, Fulgoromorpha, Derbidae). ZooKeys 825: 55–69. 10.3897/zookeys.825.31542 PMC6391365 30820160

[B10] Szwedo J (2006) First fossil record of Cedusini in the Eocene Baltic amber with notes on the tribe (Hemiptera: Fulgoromorpha: Derbidae). Russian Entomological Journal 15(3): 327–333.

[B11] Yang CT Wu RH (1994) Derbidae of Taiwan (Homoptera: Fulgoroidea). Cheng Chung Shu Chü, T’ai-pei, 230 pp.

